# Stochastic population forecasts based on conditional expert opinions

**DOI:** 10.1111/j.1467-985X.2011.01015.x

**Published:** 2012-04

**Authors:** F C Billari, R Graziani, E Melilli

**Affiliations:** Bocconi UniversityMilan, Italy

**Keywords:** Conditional expert opinions, Italy, Official population projections, Random scenario, Stochastic population forecasting

## Abstract

The paper develops and applies an expert-based stochastic population forecasting method, which can also be used to obtain a probabilistic version of scenario-based official forecasts. The full probability distribution of population forecasts is specified by starting from expert opinions on the future development of demographic components. Expert opinions are elicited as conditional on the realization of scenarios, in a two-step (or multiple-step) fashion. The method is applied to develop a stochastic forecast for the Italian population, starting from official scenarios from the Italian National Statistical Office.

## 1. Introduction

Population forecasts are crucial ingredients in long-range planning, both for government and for private institutions (see, for example, Smith (1987) and Bongaarts and Bulatao (2000)). Future trends in total population size and in age structure are of central interest to a wide range of analysts, including policy makers, scientists and planners in the public and/or private sector. For instance, trends in population by age are needed to forecast the demand for education, and to plan the provision of education at all levels. Similarly, forecasts of demographic dependence ratios are essential to the design and reform of social security systems. In the past, most population forecasts were based on relatively simple mathematical extrapolations of current trends, giving rise to so-called scenario-based population projections. Global as well as national population projections have been produced following this approach by various agencies. For instance, the United Nations, the World Bank, the US Census Bureau and Eurostat have provided world and/or regional level projections. National statistical offices have, as a routine task, produced national level forecasts. All scenario-based forecasts are produced by developing several variants for each of the three main determinants of demographic change (i.e. fertility, mortality and migration). These variants are subsequently combined through the so-called cohort–component method, usually resulting in three main projections, which are known as low, medium and high scenarios. The high–low interval is generally interpreted as containing likely future population values.

Projections are inevitably uncertain. One source of uncertainty is the lack of perfect knowledge of the population at the starting point of the forecasting time interval; this is usually a minor problem in countries with an advanced system of population statistics. The most important source of uncertainty is the unpredictability of facts and events that can influence future trends in fertility, mortality and migration. As Keyfitz (1996) remarked,

‘the best demographers provide forecasts, but none would stake their reputation on the agreement of such forecasts with the subsequent realizations’.

However, if uncertainty cannot be avoided, it must and can be measured in some way. In the scenario-based projections that are most frequently used by official agencies, uncertainty is only qualitatively accounted for. As observed by Booth (2006), the main difficulty with scenario-based projections is that it turns out to be impossible

‘to specify the probability for the high-low interval, even if probabilities could be specified for the range in component variants’

and

‘high-low intervals for population size will be inconsistent with high-low intervals for other characteristics of the projected population’.

Alho and Spencer (1985) and Lee and Tuljapurkar (1994), for instance, discussed these and other kinds of inconsistencies with scenario-based projections. Traditional population projections are, in fact, non-stochastic expert-based ‘what if?’ scenarios.

More recently, stochastic (or probabilistic) population forecasting has received great attention by researchers. The main reason for the development of stochastic forecasting methods lies in the awareness that only in this way can forecasting uncertainty be fully and coherently managed. Still, stochastic population forecasting has not yet influenced most official forecasting agencies (Lutz *et al.*, 1997; Booth, 2006), although a more probabilistic orientation has been repeatedly advocated during at least the last three decades (Land, 1986). This relative lack of influence of methodological proposals on actual official forecasting by statistical offices suggests that the possibility of linking stochastic population forecasting to classical scenario-based population projections might be a key to bridge the gap.

As already observed, virtually all population forecasts, based either on the traditional scenario approach or on the stochastic approach, adopt the standard cohort–component method (Preston *et al.*, 2001), which is characterized by two steps. In the first step, the future trajectories of the three key components of demographic change are forecast over the time horizon of interest. This first step often entails a forecast of summary indicators for these components which are then used to derive the full set of age-specific quantities (usually, rates for fertility and mortality, and absolute numbers for migration). In some cases, age-specific quantities are directly forecast. In the second step, component-specific forecasts are combined and applied to an initial population to obtain the actual population forecast, giving a full probability distribution of forecasts in the stochastic case.

In stochastic population forecasting, three main approaches have been followed to derive the probability distribution of forecasts (Keilman *et al.*, 2002). The first approach is based on time series models, which are the most classical way to address forecasting problems. For each indicator to be forecasted, a more or less complex time series model is fitted to past data, and forecasts are derived by extrapolation based on the estimated parameters. In the literature, both classical (i.e. frequentist) and Bayesian time series methods for population forecasting have been proposed (see, for example, Land (1986), Lee and Carter (1992), Lee and Tuljapurkar (1994), Hyndman and Booth (2008), Alkema *et al.* (2008, 2010), Raftery *et al.* (2009), Bijak and Wisniowski (2010) and Chunn *et al.* (2010)). A Bayesian approach makes it possible to incorporate information coming from any extra-sample source in the forecast. In particular, expert opinions can be used in the specification of the prior distribution to be assigned to the parameters of the model; see in particular Bijak and Wisniowski (2010). The best-known approach using (classical) time series models is that of Lee and Carter (1992) (see also Tabeau (2001)), which was originally proposed to forecast mortality, and later modified to address fertility forecasting (see Lee (1993) and Lee and Tuljapurkar (1994)). The Lee–Carter approach is based on a log-linear model for age-specific rates of mortality, and it includes a time-dependent mortality index modelled as a random walk with constant drift. As observed by Booth *et al.* (2005) and Bell (1997), the Lee–Carter method has been found to perform quite well for some developed countries, but its implementation, requiring data on age-specific rates of death, can be difficult for many developing countries (Li *et al.*, 2004). This is a general problem with a time series approach for developing countries, as knowledge of the past is often limited. Moreover, Lee and Miller (2001) found systematic underestimates of life expectancy by using Lee–Carter forecasts also for some developed countries. There are attempts to overcome the lack of available data by resorting to a Bayesian approach. Girosi and King (2008) used a Bayesian hierarchical model to model the dependence of age-specific rates of mortality (on a logarithmic scale) on a set of covariates and suggested a method to assign highly informative priors on the coefficients. Also Chunn *et al.* (2010) proposed an approach to life expectancy forecasting for all countries in the world based on Bayesian time series models. They used a random-walk model with non-constant drift; the latter is a non-linear function of current life expectancy and aims to describe different rates of increase in life expectancy for different countries. A Bayesian hierarchical model is used, with parameters estimated by using both past data and prior information; the prior distribution is specified by using United Nations deterministic scenarios together with additional assumptions (see Oeppen and Vaupel (2002)). Alkema *et al.* (2010) proposed a related method to forecast total fertility rates by using a fully Bayesian time series approach.

The second approach to stochastic forecasting is based on the extrapolation of empirical errors, with observed errors from historical forecasts used in the assessment of uncertainty (e.g. Stoto (1983)). In particular, Alho and Spencer (1997) proposed in this framework the so-called scaled model of error. Forecasts are obtained by adding shocks to fertility and mortality age-specific rates (expressed in logarithmic terms). The variance and correlation across age and time of shocks is estimated on the basis of the past forecast errors time series. The scaled model of error is implemented in the simulation program PEP (‘program for error propagation’; see Alho and Spencer (2005)). This approach was used for deriving stochastic population forecasts within the ‘Uncertain population of Europe’ (UPE) project (see Alders *et al.* (2007) for a description of its application in the UPE project). The approach has been applied to aggregate in a consistent way national level forecasts by Alho (2008), and to derive household level stochastic forecasts by Alho and Keilman (2010).

The third approach,which is known as the ‘random-scenario’ approach, is based on the use of expert opinions in the definition of probability distributions for the future values of summary indicators. These are then used for the subsequent production of probabilistically coherent population forecasts in the traditional cohort–component framework. Generally, this approach proceeds through the specification by an expert of high, central and low values of summary indicators for each component at the end point of the forecast interval; these values are assumed to correspond to prediction intervals of some given coverage.

The use of experts in probabilistic forecasting is not a prerogative of the random-scenario approach. As noted earlier, Bayesian time series models can involve expert opinions in the elicitation of prior distributions; see for instance Bijak and Wisniowski (2010). Similarly, methods that extrapolate past forecast errors can require the intervention of experts; indeed, these errors are added to point predictions, which are usually derived from central scenarios of a traditional deterministic forecast, and these scenarios in turn come from experts’ evaluations.

Some contributions in the literature have explicitly compared different approaches to population forecasting. The random-scenario approach is, as observed by many scholars, more appealing to official forecasters, owing to its simplicity, its framework based on scenarios and the direct involvement of experts (see, for instance, the comments by Goldstein (2004) and (Lutz and Goldstein (2004)). Lutz (1996) argued that the use of expert opinions allows us to take into account behavioural theories on the future of population. Indeed, expert opinions might be valuable in embedding knowledge on trends (e.g. policy changes and environmental change) that are assumed to have an effect on population dynamics and that have also often been left out of the picture (Cohen, 2003). The impossibility of taking into account expected trends in factors affecting demographic change is probably the main weakness of the pure (classical) time series approach and of all methods that are essentially based on extrapolation of past trends. An obvious main advantage of expert-based forecasting is that it does not require (at least directly) data on the past; for this reason, this approach can be especially useful for developing countries, for which past data are usually poor.

The literature has also outlined the weaknesses of expert-based forecasts. The main criticism is related to the well-known and widely observed tendency of experts to underestimate uncertainty. Keilman (1990) observed that, particularly when recent trends have been stable, the overconfidence of experts results in overly narrow prediction intervals. Lutz *et al.* (1999, 2004) addressed this problem by placing greater emphasis on expert arguments rather than opinions in developing scenario-based forecasts. The conservativeness of expert opinions with respect to decline in mortality is widespread; Alho and Spencer (1990) for instance showed that US experts systematically forecast a smaller decline in mortality than actually occurred. Also, for what concerns fertility, expert opinions have not been accurate (see Lee (1993) and Booth (2006)). A further drawback of expert-based forecasting is its tendency to be limited to summary measures, so details in age-specific rates are often missing. Moreover, correlations over time and between sexes (for a single indicator) and correlations between different indicators must be elicited directly from experts, who are not at ease with interpreting such quantities. Indeed, correlations between pairs of quantities are difficult to elicit directly. Finally, existing random-scenario methods, being generally based on trajectories that are obtained by the interpolation of a starting (known) and a final (random) value, are characterized by a variance and covariance structure which is not particularly flexible. In particular, when the interpolation is linear, correlations over time are by definition all equal to 1.

In this paper, we introduce a method that lies in the framework of the random-scenario approach and that builds on the definition of a flexible stochastic process, while retaining a basic simplicity and an immediate interpretation of the expert opinions on which the definition of the process is based. Roughly speaking, the stochastic population forecasting method that we propose proceeds through a series of subsequent expert-based conditional evaluations on the future of demographic components, expressed through summary indicators, given the values of the indicators at previous time points. Conditional evaluations given by the expert are in the traditional form of medium–low–high scenarios. When the coverage of these scenarios in terms of probability is not specified *a priori*, or when we need to address experts’ overconfidence, appropriate coverage values for such scenarios might be chosen: a greater reliability of an expert will result in a higher coverage and vice versa. In our example, using average historical errors and adopting the assumption that uncertainty in the future will be virtually of the same magnitude of that in the past, we consider a cautious choice of coverage of about 50%. This means, in other words, that low and high (conditional) values given by the expert are assumed to correspond to quartiles of the (conditional) distributions. Assigning a Gaussian joint distribution to the values of the indicators, at some string of equally spaced time points (i.e. splitting the forecasting period), such evaluations are then used to derive the parameters of the probability distribution of the forecast. By interpolation, the process is extended over the whole forecast time span. Indicators are then converted to age-specific rates and the complete forecast is derived by using a starting population. In our proposal, variances and serial correlations, correlation between sexes and (possibly) between indicators are derived from the simple and easily interpretable expert opinions and need not be directly elicited by experts. At the same time, they can assume a wider range of values than in random-scenario approaches that have been developed so far. Indeed, whereas in standard applications of the random-scenario approach the path of each component is a random line, here it turns out to be a more general trajectory and then allows for non-unitary correlations over time. Our method is implemented by using MATLAB code (the program is freely available on request from the author for correspondence).

The remainder of this paper is structured as follows. Section 2 is devoted to a description of the method, based on the simple treatment of a single indicator. In Section 3, the implementation of the method is described. In Section 4, a full-fledged example at the national level, for Italy, is developed, starting from existing scenarios provided by official statistical agencies. In Section 5, a discussion of some relevant issues starting from experiments in the example is provided. Section 6 includes a short summary and discussion. Appendix A provides technical details on the method proposed.

## 2. Approach proposed

In this section we illustrate the method for the derivation of the predictive distribution of summary demographic indicators based on conditional expert opinions. We start from the case of a single generic indicator to generalize it to a (limited) set of standard indicators. First, we introduce some simplifying assumptions to limit the potentially huge number of parameters. We assume that the initial population is known, so that forecast uncertainty depends only on the uncertainty over the dynamics of the components of demographic change. Moreover, also to be consistent with the idea of using the opinion of experts, we summarize demographic components through a standard series of indicators: the total fertility rate TFR and mean maternal age at birth MAB for fertility; male and female life expectancy at birth EM and EF respectively for mortality; male and female net migration flows MM and MF respectively for migration. Although one could in principle separate immigration and emigration without particular problems in the model, net migration is here used for simplicity. In line with the literature on developed countries (see, for example, Booth (2006)) we also assume that the demographic components are independent (i.e. the pairs (TFR, MAB), (EM, EF) and (MM, MF) are mutually independent). TFR and MAB are assumed to be independent, whereas we allow for correlation between sexes for mortality and migration. In a subsequent stage, age-specific quantities (i.e. age-specific rates for fertility and mortality, and age-specific net migrants for migration) are derived by using parametric or semiparametric models, so that probability distributions of age-specific quantities are obtained from the (joint) distributions of indicators.

We now describe the procedure in a more formal fashion. We first consider the case of a single summary indicator, which is denoted by *R*. Then, we consider the more general case, taking into account the joint distribution of age-specific quantities, with the mutual dependence–independence structure described above.

Let [0,*T*] be the forecasting interval and let *t*_1_ be an inner point that splits it into two subintervals (for instance, but not necessarily, *t*_1_ could be the midpoint *T*/2). For notational convenience, let *t*_0_=0 and *t*_2_=*T*. It is worth emphasizing here that the method can be generalized, without major complications, to more than two subintervals. Our purpose is to define a Gaussian random vector (*R*_*t*1_,*R*_*t*2_) from which, by linear interpolation starting from *R*_0_, the full random process (*R*_*t*_,*t* ∈ [0,*T*]) is derived. In our framework, the five conditions to be imposed to fix the parameters (two means, two variances and the correlation) of the distribution of (*R*_*t*1_,*R*_*t*2_) are determined on the basis of conditional expert opinions. We illustrate the procedure step by step.

At the first step, the expert is asked to express central and high scenarios for the indicator *R* at the time point *t*_1_. These quantities are used to define the expected value and the variance of the random variable *R*_*t*1_. As we are also interested in starting from official population forecasts, central and high scenarios might also be those provided by official forecasting agencies in traditional scenario-based projections. Denoting by *c*_1_ and *h*_1_ respectively the central and high scenarios for *R*_*t*1_, we set *E*(*R*_*t*1_)=*c*_1_ and 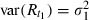, where 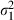 is such that *h*_1_ is the quantile of some (sufficiently high) order of the normally distributed random variable *R*_*t*1_. Hence


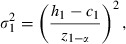


*z*_1−*α*_ being the quantile of order 1−*α* of the standard normal distribution.

At the second step, the expert is asked to provide the values of the indicator that represent (conditional) central scenarios at time *t*_2_ given that, at time *t*_1_, the indicator values are equal to respectively the central and high scenarios fixed in the previous step. More precisely, the expert is required to elicit the values of the indicator by answering the following two questions:

- ‘What would in your opinion be a central scenario value for the indicator *R* at time *t*_2_, if at time *t*_1_ the indicator reaches *c*_1_?’, and
- ‘What would in your opinion be a central scenario value for the indicator *R*, at time *t*_2_, if at time *t*_1_ the indicator reaches *h*_1_?’.

The values elicited by the expert (let us call them *c*_2_|*c*_1_ and *c*_2_|*h*_1_ respectively) are considered as the conditional means of *R*_*t*2_ given that *R*_*t*_ is equal respectively to its mean or to its quantile at the previous time point, i.e. *c*_2_|*c*_1_=*E*(*R*_*t*2_|*R*_*t*1_=*c*_1_) and *c*_2_|*h*_1_=*E*(*R*_*t*2_|*R*_*t*1_=*h*_1_). Clearly, since (*R*_*t*1_,*R*_*t*2_) is assumed to be a Gaussian vector, the first of these conditional means is equal to the unconditional mean. Note that, up to now, we have completely defined the marginal distribution of *R*_*t*1_ (in the first step) and the conditional mean of *R*_*t*2_ given *R*_*t*1_ (in the second step).

At the third step, we elicit from the expert, through a question similar to the previous questions, the high scenario *h*_2_|*c*_1_ for the indicator at time *t*_2_ if *R*_*t*1_ reaches *c*_1_, i.e. we ask the expert for the quantile (of some order 1−*α*) of the conditional distribution of *R*_*t*2_ given *R*_*t*1_=*c*_1_. This value (combined with the previous values) yields the conditional variance of *R*_*t*2_ given *R*_*t*1_ (which turns out to be, under the normality assumption, constant with respect to the value of *R*_*t*1_). A variant of the method replaces the last step by the following assumption: the conditional variance of *R*_*t*2_ given *R*_*t*1_ is equal to 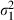, the marginal variance of *R*_*t*1_, i.e. var(*R*_*t*2_|*R*_*t*1_)=var(*R*_*t*1_). Clearly this assumption is reasonable for equally spaced time points, i.e. when *t*_1_=*T*/2; in the more general situation of subintervals of different lengths, the hypothesis must be modified assuming that


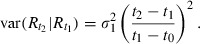


Having obtained, in the second and third steps, the conditional mean and the conditional variance of *R*_*t*2_ given *R*_*t*1_, the conditional distribution is fully characterized, and the distribution of the random vector (*R*_*t*1_,*R*_*t*2_) is defined. As we have already said, the distribution of the whole process (*R*_*t*_,*t* ∈ [0,*T*]) is obtained through simple linear interpolation of *R*_0_, *R*_*t*1_ and *R*_*t*2_. In Appendix A, the explicit expressions of the parameters of the joint distribution of (*R*_*t*1_,*R*_*t*2_) are derived as functions of the expert opinions.

Here it is worth making some remarks on the characteristics of the random process that we have generated for the single indicator *R*.

**Remark 1.** As shown in Appendix A, the correlation coefficient is either positive or negative depending on the difference *c*_2_|*h*_1_−*c*_2_|*c*_1_. More precisely, the correlation coefficient is positive if the value of the central scenario provided by the expert at time *t*_2_ is higher under the hypothesis that the high scenario is reached at time *t*_1_. As an example, this would happen if experts assume that for example decreases in mortality are cumulative. The opposite (a negative correlation) might happen for example if an expert assumes that having a high net migration at time *t*_1_ will cause a rebound at time *t*_2_. The correlation coefficient is equal to 0 when the value of the indicator at time *t*_1_ has no effect on the expert evaluation at time *t*_2_, i.e. *R*_*t*1_ and *R*_*t*2_ are independent. Moreover, the correlation coefficient is necessarily strictly lower than 1, unless the high and central scenarios at time *t*_2_ coincide (but this would imply a null variance).

**Remark 2.** It can be easily proved that the variance of the indicator *R*_*t*_ turns out to be an increasing function of *t*. If the last step of the procedure, which is needed to determine the conditional variance, is replaced by the assumption


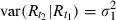


(possibly modified when intervals have unequal lengths), then the parameters 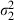 and *ρ* are generally different from the previous parameters; their explicit expressions are given in Appendix A. Also in this case it is easy to verify that the variance of *R*_*t*_ is an increasing function of the time *t*.

**Remark 3.** Our procedure determines the parameters (i.e. the moments) of the Gaussian vector by means of the conditional evaluations of the expert, which are essentially conditional quantiles (medians and upper quantiles). Indeed, in our opinion, quantiles are of more direct and easy interpretation and elicitation than moments.

**Remark 4.** Possible generalizations of the method concern the choice of the distribution and the way that interpolation is performed. At the cost of greater complexity, the Gaussian distribution can be replaced by skew normal (Azzalini and Dalla Valle, 1996) or multivariate gamma (Kotz *et al.*, 2000) distributions. With these assumptions we could, for instance, take into account the possible asymmetry or tail heaviness of the distribution of the indicator. Additional information elicited from the expert (i.e. lower besides higher scenarios) can help to decide whether the normal distribution is a good choice or not. For what concerns the interpolation of generated values, we could of course replace the linear approach that we used with a non-linear approach (for instance quadratic or, more generally, polynomial). Also in this case, there is a trade-off between conceptual and computational simplicity and fitting ability.

**Remark 5.** Our proposal can easily be implemented by using the inputs that come from more than one expert. In the case of more than one expert, we first need to combine expert opinions (for instance, simply by averaging them), and then we can use the obtained values as input for the definition of the stochastic process following the procedure outlined above.

**Remark 6.** The high scenarios elicited from the expert can be interpreted as quantiles of any order (or, the expert can be directly asked to refer to a quantile). It is widely recognized that experts tend to underestimate uncertainty. To take into account experts’ overconfidence, we can assume that the high–low intervals that are provided by experts have a low coverage. For instance, in the example that follows, we assume a 50% coverage probability. Such a kind of choice is in line with the observed uncertainty of the past historical forecasts.

Let us now consider the general setting in which the joint distribution of all indicators is needed for the pair of future time points. Since we use six indicators (TFR, MAB, EM, EF, MM and MF), we need the distribution of a random vector of dimension 12. Even if the goal can appear, at first glance, very difficult and the task may seem quite daunting, it is possible to define the required multi-dimensional distribution by assuming that some components are mutually independent, by retaining the normality assumption and by proceeding, as we have done for a single indicator, in a conditional way. In fact, because of the independence between demographic components and the assumption that TFR and MAB are independent, we must define only the following two-dimensional and four-dimensional vectors:


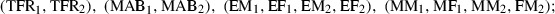


as usual, indices refers to time points. For the first (two-dimensional) vectors we can proceed exactly as we have done earlier. For each of the four-dimensional vectors, all the parameters (means, variances and correlations) are elicited from the expert as ‘conditional scenarios’, answering to questions such as ‘what would be the central scenario value for the males indicator *R* at time *t*_2_ given that the males indicator *R* at time *t*_1_ reaches *c*_1_ and the females indicator at time *t*_2_ reaches *c*_2_?’. The explicit step-by-step procedure is described in Appendix A.

Clearly, once the overall joint distribution of the indicators, by sex and by time, has been defined, linear interpolations for each indicator complete the characterization of the whole random process.

**Remark 7.** It is worth emphasizing that, by proceeding in the definition of the multivariate normal distribution through conditional evaluations, we have the the advantage of ensuring the coherence of all parameters. In particular, the covariance matrix is surely positive definite when obtained this way. In contrast, a direct choice of correlations and variances could in principle result in non-admissible values.

## 3. Implementation of the proposal

We now describe the algorithm for implementing the proposal that was described in the previous section to derive a full set of age- and sex-specific stochastic population forecasts. The actual implementation makes use of a specially designed program developed in MATLAB where actual forecasts are obtained through simulation. The male and female populations are separately forecasted, grouped into age intervals. The forecasting period is divided into time intervals that have the same length as the age intervals. For each forecasting interval, the algorithm follows the cohort–component method, i.e. it

- projects forward the population for each age group, by applying age- and sex-specific survival rates derived from life expectancy,
- computes the number of births for each subgroup over the time interval and the number of births still alive at the beginning of the next interval by using fertility and mortality indicators and
- adds net migration flows and projects forward the number of immigrants and of births from immigrants who are still alive at the beginning of the next time interval.

The algorithm requires the following inputs:

- the starting population by age and sex,
- the length of the time intervals dividing the forecasting period (equal to the length of the age intervals),
- the (conditional) scenarios (central and high) provided by the expert(s) on all summary indicators,
- the level of the quantiles elicited from the expert(s),
- the confidence level of the predictive intervals of the forecast generated and
- the number *N* of samples to draw for each component from the corresponding multivariate Gaussian distribution.

The algorithm works through the following four steps. Let *k* be the number of time points dividing the forecast interval (in the example we shall consider *k*=2).

At the first step, the parameters of the Gaussian multivariate distribution of each component vector associated with the *k* points are derived from the conditional scenarios. In the program, it is possible to specify whether the *k* intervals that are determined by such points are equally or unequally spaced.

At the second step, for each component, *N* samples are drawn from the corresponding multivariate Gaussian distribution. The values of the single indicator at each time point of the forecasting interval are obtained by linear interpolation. Therefore, at this step, we obtain, for each summary indicator, *N* draws from the joint distribution of the values at each of the points in the forecasting interval.

**Fig. 1:**
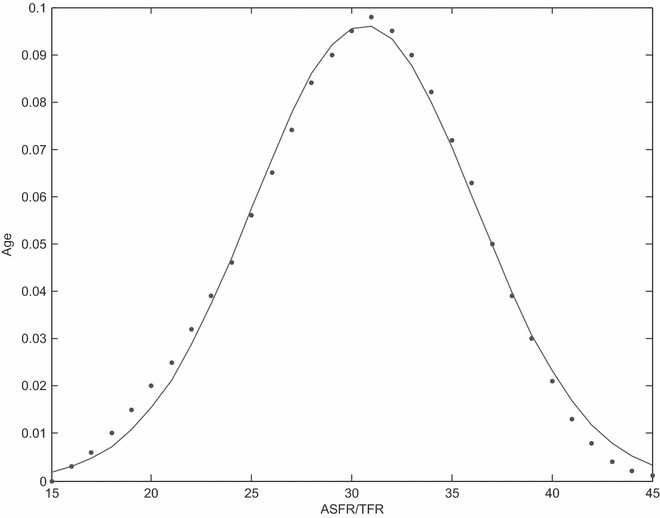
Italian 2004 age-specific fertility rates fitted with a rescaled normal distribution

At the third step, the whole set of age- and time-specific quantities is derived starting from the summary indicators. In this implementation, we chose extremely simple ways to obtain smooth age-specific quantities starting from summary indicators. *N* matrices of male and *N* matrices of female age and time-specific age-specific mortality rates are derived from the corresponding vectors of male and of female life expectancies at birth. For each time point, age-specific mortality rates are obtained from the corresponding life expectancies at birth on the basis of life tables generated through a semiparametric approach, using standard well-known *Brass logit* models (Brass (1971); see also, for example, Vassin (1994) and Booth (2006)). *N* matrices of age- and time-specific fertility rates are derived from the vectors of total fertility rates and the vectors of mean maternal ages at birth. Age-specific fertility rates ASFR_*t*_(*x*) are obtained from the total fertility rates TFR_*t*_ and mean maternal ages at birth MAB_*t*_ on the basis of the following rescaled normal model:


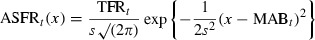


where the standard deviation *s* is obtained by fitting the same curve to the age-specific fertility rates at 2004 and it is held fixed for the entire projection period. Whereas many more complex models have been proposed for the age patterns of fertility (e.g. Booth (2006)), and could be used in our implementation, we chose the normal distribution for its simplicity. For example, Fig. 1 displays the 2004 Italian age-specific fertility rates along with the fitted curve.

*N* matrices of male and *N* matrices of female age-specific net migration flows are derived from the corresponding vectors of total net flows, using a rescaled gamma model. This is a simplifying assumption that assumes the absence of preschool, retirement and post-retirement peaks in the age profile of migrations, the only peak being related to labour migration. Moreover, modelling total net flows assumes that the distribution of in migration and emigrations is proportional over the age range. More general models of migration, both in terms of converting total flows to age-specific flows, and in terms of separating immigrations and emigrations could also be implemented.

At the fourth and last step, the matrices of age-specific mortality rates by sex and the matrices of age-specific fertility rates along with the matrices of the migration flows by age and sex are used as inputs of the cohort–component model to derive *N* matrices of the total population by age and time for each sex. Such matrices can be considered as draws from the corresponding distribution of the male and female population, so that forecasts and confidence intervals can be derived from them. For instance, the forecast of the female population size of a fixed age and for a fixed forecasting time is given by the arithmetic mean of the *N* corresponding draws of the female population size for the age and forecasting time considered. An estimate of the 90% confidence interval is given by the interval determined by the 0.05*N*th and 0.95*N*th sample quantiles. One can obtain stochastic forecasts for summary indicators related to the age structure of the population (e.g. dependence ratios) this way also.

## 4. An application: stochastic population forecasts for Italy to 2050 derived from official projections

In this section, we describe the application of our method to derive stochastic population forecasts for Italy (in a baseline example), with the time horizon 2009–2050. More specifically, we develop population forecasts by single year of age (considering 101 age intervals, from 0–1 to over 100 years) starting from official projections, i.e. treating the scenarios of the projections as expert opinions. We also derive probabilistic forecasts of summary indicators on the age structure of the population.

### 4.1. Input data for the baseline example

Forecasts are derived by using as expert inputs the scenarios on indicators provided by the Italian National Statistical Institute ISTAT the official statistics agency of Italy (Italian National Statistical Institute, 2008). Using the approach outlined before, we can therefore provide a probabilistic version of official population projections. Our reliance on ISTAT scenarios does not make it possible to elicit the correlation between sexes. In the baseline application, we therefore assume that life expectancies for males and females on one side and net migration flows for males and females on the other side are independent.

We divide the forecasting interval into two subintervals, 2009–2030 and 2030–2050, and for each indicator *R* we associate with the two points *t*=2030 and *t*=2050 a bivariate Gaussian random vector with parameters obtained on the basis of the following assumptions, using the notation that was introduced in Section 2:

- *c*_1_=*E*(*R*_2030_) is set equal to the central ISTAT scenario at time *t*=2030;
- *h*_1_ is set equal to the high ISTAT scenario at time *t*=2030 and is assumed to be the quantile of order 0.75 of the distribution of the indicator;
- *c*_2_|*c*_1_=*E*(*R*_2050_|*R*_2030_=*c*_1_)=*E*(*R*_2050_) is set equal to the central ISTAT scenario at time *t*=2050;
- *c*_2_|*h*_1_=*E*(*R*_2050_|*R*_2030_=*h*_1_) is set equal to the high ISTAT scenario at time *t*=2050.

The variance of the indicator at time 2030 is computed from the first two assumptions, whereas, to derive the variance of the indicator at 2050, we follow the second method described in Section 2 and set var(*R*_2050_|*R*_2030_)=var(*R*_2030_)(20/21)^2^. The constant (20/21)^2^ is because the forecasting period is divided into two subintervals of different length.

Table 1 displays the ISTAT scenarios for the various indicators: migrations of males and females are expressed in thousands.

**Table 1:** ISTAT scenarios for indicators†

*Parameter*	*Results for the following years:*		
	
	*2009*	*2030*	*2050*
*μ*(TFR)	1.33	1.57	1.58
*q*(TFR)		1.70	1.75
*μ*(MAB)	30.32	32.60	33.40
*q*(MAB)		32.70	33.60
*μ*(EF)	83.71	87.50	89.50
*q*(EF)		89.10	91.60
*μ*(EM)	77.92	82.20	84.50
*q*(EM)		84.00	86.80
*μ*(FM)	158.896	101.150	101.789
*q*(FM)		124.043	122.982
*μ*(MM)	147.853	94.027	94.710
*q*(MM)		115.423	114.435

†TFR is the total fertility rate, MAB is the mean maternal age at birth, EF and EM are respectively life expectancy at birth for females and males, and FM and MM are respectively net migration flows in thousands. for females and males. *μ* and *q* are respectively the central and high scenario value.

Table 2 shows the means, standard deviations and correlation coefficients for the six processes that we obtain in the baseline example. The correlation coefficient is positive, given the way that we handled the transformation of scenarios into conditional expert opinions.

**Table 2:** Means, standard deviations and correlations of indicators used to derive the probabilistic forecast

*Indicator*	*μ*_2030_	*μ*_2050_	*σ*_2030_	*σ*_2050_	*ρ*
TFR	1.57	1.58	0.16	0.29	0.808
MAB	32.60	33.40	0.14	0.32	0.903
EF	87.50	89.50	2.717	3.846	0.802
EM	82.20	84.50	2.669	4.253	0.809
FM	101.050	101.789	33.941	45.080	0.697
MM	94.027	94.714	31.722	42.047	0.689

### 4.2. Results of the baseline example

**Fig. 2:**
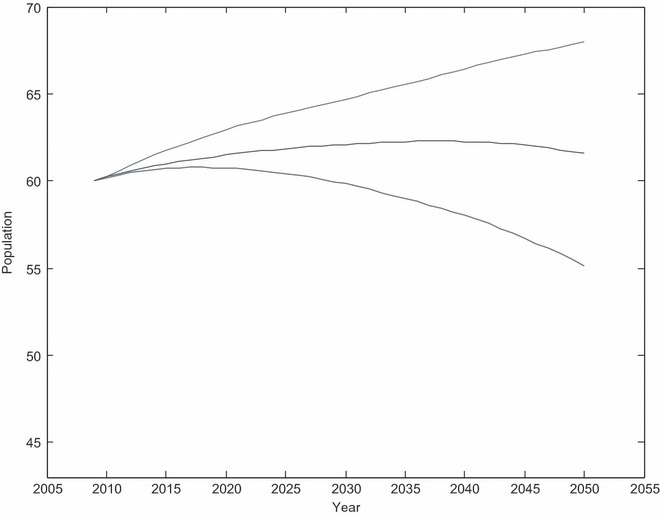
Italian population forecasts and 90% confidence intervals (in millions) based on ISTAT scenarios (2009–2050)

Fig. 2 displays the forecast of the Italian total population for 2009–2050, whereas Table 3 shows the 2030 and 2050 total population forecast and the elderly dependence ratio forecast, along with their 90% confidence intervals As concerns the absolute values for the total population, scenarios are consistently projecting a growing population on average. Population growth is likely up to 2030: the starting population is 60.0 million at the beginning of 2009, and the mean for 2030 is 62.1 million, the lower bound and upper bound being respectively 59.8 million and 64.7 million). This could be compared with ISTAT non-probabilistic projections, for which the central scenario at 2030 also results in a total population of 62.1 million (the low scenario is 59.7 million and the high scenario 64.4 million). For 2050, as expected there is more uncertainty, with a mean of 61.6 million, and a decrease in the total population which is also within the confidence interval (between 55 million and 68 million). Again, this could be compared with ISTAT projections, for which the central scenario at 2050 is 61.7 million (the low scenario being 55.9 million and the high scenario being 67.2 million).

**Table 3:** Italy: forecast and 90% confidence intervals for 2030 and 2050 total population and elderly dependence ratio

*Year*	*Total population (millions)*	*Elderly dependence ratio (%)*
2009	60.045	32.20
2030	62.100 (59.819, 64.675)	46.13 (43.21, 50.44)
2050	61.559 (55.083, 68.009)	65.52 (56.39, 76.87)

When we analyse the elderly dependence ratio (the population aged more than 65 years over the population aged between 14 and 65 years, as a percentage), there is virtually no uncertainty in the fact that the Italian population will continue to age during the whole interval. Compared with 32.2% in 2009, the elderly dependence ratio is projected to rise to 46.1% in 2030 (with confidence intervals ranging from 43.2% to 50.4%) and to 65.5% in 2050 (with confidence intervals ranging from 56.4% to 76.8%). In the official ISTAT projections, the central scenario for 2030 is 43.6% (with the low scenario at 42.3% and the high scenario at 44.8%). At 2050, ISTAT projects 60.9% for the central scenario, 61.9% for the high scenario and 59.5% for the low scenario. The uncertainty of our forecasts to 2050 might have implications for forecasts that are influenced by population aging (e.g. the pension system and healthcare costs). Indeed, our forecast could provide a stochastic input for forecasts that take population as a driving factor.

## 5. Evaluation of the baseline example

This section provides an evaluation of our baseline example, including some extensions and robustness checks. More specifically, we introduce the correlation between sexes, we develop a counterfactual scenario without migration and we compare the results of our forecasts with those obtained through an alternative probabilistic population forecasting approach: the scaled model of error.

### 5.1. Correlation between sexes

In the baseline example we assume that the pairs of indicators (EM, EF) and (MM, MF) are mutually independent. We could, however, expect that explicitly considering the correlation between sexes for mortality and migrations would increase the variability of the forecast. Therefore, since it is not possible to use scenarios provided by ISTAT to elicit such correlations (although this elicitation is in general feasible with our method), we derive forecasts by using the same inputs as in the example for the marginal distributions at each time point and imposing a correlation of 0.8 between sexes. Results are shown in Table 4. Compared with the baseline example, we indeed observed an increased variability in forecasts. At the 2050 horizon, the size of the interval increases from 12.9 million to 15.7 million for the total population, and from 20.5% to 25.6% for the elderly dependence ratio.

**Table 4:** 2030 and 2050 total population and elderly dependence ratio assuming a 0.8 correlation between sexes: forecasts and 90% confidence intervals

*Year*	*Total population (millions)*	*Elderly dependence ratio (%)*
2009	60.045	32.20
2030	62.108 (59.537, 65.499)	46.17 (42.73, 53.13)
2050	61.542 (54.020, 69.819)	65.70 (54.51, 80.00)

### 5.2. The no-migration case

Results of the forecasts in the baseline example depend crucially on migration, which has been the (vastly unforeseen) major driver of Italian population dynamics during recent decades. The Italian population had been predicted to shrink by all forecasting agencies (Billari and Dalla Zuanna, 2008) whereas the starting point of our forecast, at 2009, is the highest population ever recorded. For this reason, and to assess the importance of migratory flows in forecasting the Italian population, we also run a zero-migration forecasting scenario (similarly to what is often done by statistical agencies).

**Fig. 3:**
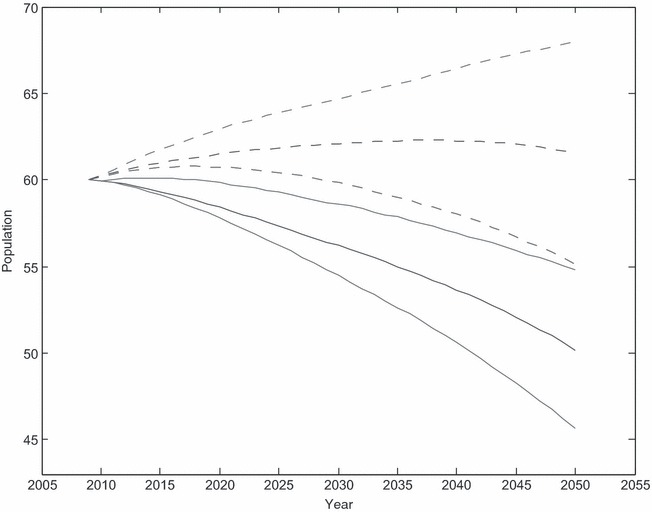
Italian population forecasts and 90% confidence intervals (in millions) (2009–2050), without migration (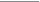) and with migration (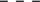)

Fig. 3 displays the forecasts of the Italian total population for 2009–2050 based on ISTAT scenarios, distinguishing two cases according to whether migratory flows are assumed to be absent or present. Tables 5 and 6 display the 2030 and 2050 total population forecasts and elderly dependence ratios forecasts along with the confidence intervals, for the two cases. These results confirm the high sensitivity of population forecasts, and in particular of indicators related to population aging, to the forecast of the migration component.

**Table 5:** 2030 and 2050 total population forecasts and 90% confidence intervals, without and with migration

*Year*	*Total population without migration (millions)*	*Total population with migration (millions)*
2009	60.045	60.045
2030	56.191 (54.448, 58.582)	62.100 (59.819, 64.675)
2050	50.175 (45.627, 54.767)	61.559 (55.083, 68.009)

**Table 6:** 2030 and 2050 elderly dependence ratio forecasts and 90% confidence intervals, without and with migration

*Year*	*Elderly dependence ratio without migration (%)*	*Elderly dependence ratio with migration (%)*
2009	32.20	32.20
2030	51.47 (48.49, 56.25)	46.13 (43.21, 50.44)
2050	78.61 (65.51, 72.91)	65.52 (56.39, 76.87)

### 5.3. Comparison with the scaled model of error

We now compare the results of our baseline example with those obtained for Italy with another model. In particular, we build a forecast based on the scaled model of error model, which is used for deriving stochastic population forecasts within the UPE project (see Alho and Spencer (1997, 2005) for a description of the method and Alders *et al.* (2007) for a description of its application in the UPE project). In our comparison, the main focus is on forecasting uncertainty. We implement the scaled model of error by using the computer program PEP that was written by Juha Alho. Using PEP, we derive the forecasts of the Italian population from 2010 to 2050, on the basis of the new forecasts of age-specific mortality and fertility rates released by the Italian National Statistical Institute (2008) and used in our scenarios (we therefore update the forecasts of the UPE project). As for the variances and correlations of the forecast error increments across ages and time for each component, we use the same assumptions as were made in the UPE forecasts. To compare our results with those obtained by using the scaled model of error, we ran our forecasts again, with the assumptions on the total fertility rate and on life expectancies for males and females as derived from ISTAT scenarios (at 2030 and 2050). The migratory flows are, instead, treated as a non-stochastic component: for each year we consider a constant net flow of female immigrants equal to 138061 and of male immigrants equal to 128769. The same gamma distribution is used to derive the age-specific immigrant flows for each year.

Table 7 shows the means and standard deviations of the total fertility rates and the life expectancies for males and females for the two methods and Table 8 shows the 2030 and 2050 forecasts of the Italian population obtained from the two methods along with the corresponding confidence intervals. Our forecasts show a lower variability compared with those obtained from the scaled model of error. Such lower variability is partially because our use of statistical agencies as experts produces a smaller variance, compared with the variance that in the scaled model is estimated from past forecast errors, as shown in Table 8. PEP forecasts are obtained by adding to the fertility and mortality age-specific rates shocks with variance and correlation across age and time estimated on the basis of the past forecast errors time series. In our approach, instead, we randomize the total fertility rate and the life expectancy for males and females at birth and derive the age-specific rates from given models.

**Table 7:** Means and standard deviations of vital rates from ISTAT scenarios and the scaled model of error

*Indicator*	*Results for ISTAT scenarios*					*Results for scaled model of error*				
		
	*μ*_2030_	*μ*_2050_	*σ*_2030_	*σ*_2050_	*ρ*	*μ*_2030_	*μ*_2050_	*σ*_2030_	*σ*_2050_	*ρ*
TFR	1.57	1.58	0.160	0.290	0.808	1.60	1.60	0.390	0.883	0.710
EF	87.50	89.50	2.717	3.846	0.802	87.80	89.90	2.075	3.674	0.850
EM	82.20	84.50	2.669	4.253	0.809	82.40	84.60	1.894	3.678	0.850

**Table 8:** 2030 and 2050 total population forecasts and 90% confidence intervals

*Method*	*Results (millions) for the following years:*		
	
	*2009*	*2030*	*2050*
Conditional opinions	60.045	62.689 (61.101, 65.489)	62.357 (58.824, 69.787)
Scaled model of error	60.045	62.100 (56.214, 68.244)	62.527 (48.050, 78.316)
Conditional opinions with scaled model of error inputs	60.045	61.985 (59.208, 64.756)	61.632 (51.228, 71.801)

To understand the reasons for the difference in the forecasts’ variability better, we also ran our forecasting model by using the means and the covariance matrices that were obtained from the scaled model of error (shown in Table 8) as inputs for total fertility rates and the life expectancies for males and females. For migration flows and the mean maternal age at birth we use the inputs derived from the conditional expert opinions. Table 8 displays the 2030 and 2050 forecasts of total population. The length of the confidence intervals is wider than the length of the intervals that were obtained on the basis of the expert evaluations, but still smaller than the length of the intervals that were obtained by applying the scaled model of error. Therefore, we can argue that the smaller variability of the forecasts obtained by applying our method is also due to the models used for the derivation of the age patterns from summary indicators.

## 6. Summary and concluding remarks

In this paper, we introduced a method that can be used to derive expert-based stochastic population forecasts through a random-scenario approach. Experts’ evaluations are elicited in a conditional way: for each time point, experts are asked to provide central scenarios given central or high scenarios at the previous time points. It is worth emphasizing that the quantiles to be elicited from experts at different times need not be of the same order. The need to consider different orders is crucial when, as in our application, high scenarios of official projections are used as conditional quantiles. Indeed as observed by one of the referees, the way that high scenarios are specified leads to predictive intervals that are too narrow in the short run and too wide in the long run; see Bongaarts and Bulatao (2000).

The method that we suggest is of general validity, as it can be applied to inputs from one or more experts (including the combination of different forecasts to obtain ‘consensus forecasts’; see Billari *et al.* (2010)), and to quite general specifications of the cohort–component model of demographic forecasts. In the example of application, we discussed how to derive a stochastic version of the official population projections for Italy which was developed by ISTAT, i.e. we treated ISTAT as the expert providing information to inform our model. Our results show that, although the Italian population is likely not to undergo sizable changes up to 2030, considerable uncertainty exists when the time horizon is extended towards 2050. There is, however, less uncertainty in the fact that the elderly dependence ratio will rise. The importance of the migration component is, however, massive in determining population dynamics.

Sensitivity analyses showed that the interpretation of experts’ opinions in terms of coverage of their scenarios is important, and that the correlation between sexes is also important (and in principle can be elicited in a conditional way). It is important here to underline some of the *caveats*, also because they suggest lines for future research. We did not explicitly survey a number of experts for our example, and the aggregation of expert opinion (i.e. the weights that are given to different experts) might be an important challenge in this case. We did not explicitly consider, in the current implementation of the method, the uncertainty in the initial age and sex distribution of the population, although it could be handled by using the same expert-based probabilistic approach. Moreover, we did not explicitly consider the uncertainty deriving from the way that summary indicators given by the experts are translated into age-specific rates or absolute numbers. The comparison between our results and those obtained through the scaled model of error seemed to suggest that this is a relevant source of uncertainty.

Despite the *caveats* that we mention, our application shows that the method we propose is easy to use. Although some limitations need further, but not overly difficult, methodological work, we believe that the approach characterizing our method makes it a candidate also for official agencies that routinely provide population forecasts.

## Appendix A

In this appendix we present some explicit computations of the parameters of the Gaussian multivariate distribution generated, in our approach, through the expert inputs. Consider first the case of a single indicator *R*, so that our interest is in the joint distribution of *R*_*t*1_ and *R*_*t*2_. Recall that, for a bivariate Gaussian vector (*R*_*t*1_,*R*_*t*2_) with means *μ*_1_ and *μ*_2_, variances  and  and correlation *ρ*, the conditional mean function is

and the conditional variance is, for any *x*,

Hence, experts’ inputs *c*_1_,*h*_1_,*c*_2_|*c*_1_,*c*_2_|*h*_1_ and *h*_2_|*c*_1_, as introduced in Section 2, give the following results.

- *μ*_1_=*E*(*R*_*t*1_)=*c*_1_ (the central scenario for the indicator at time *t*_1_).
- *μ*_2_=*E*(*R*_*t*2_|*R*_*t*1_=*μ*_1_)=*c*_2_|*c*_1_ (the central scenario for the indicator at time *t*_2_ given the central scenario at time *t*_1_).
- , where *h*_1_ is the high scenario for the indicator at time *t*_1_.
- The conditional variance var(*R*_*t*2_|*R*_*t*1_) is linked to the conditional quantile *h*_2_|*c*_1_ through the formula

Hence the remaining parameters  and *ρ* are determined as solutions of the system

(1)

i.e.

(2)

and

i.e., using the second equation in expression (1) giving the sign of *ρ*,

(3)

Following remark 2 in Section 2, we recall that, when the last step of the procedure that we are proposing is replaced by the assumption

(possibly modified as described in Section 2 when intervals have unequal lengths), then the parameters  and *ρ* are given respectively by

(4)

and

Now we describe explicitly all the steps that are needed to define the four-dimensional (Gaussian) distribution of (MLE_1_,FLE_1_,MLE_2_,FLE_2_) and (MMF_1_,FMF_1_,MMF_2_,FMF_2_) completely. To simplify the notation and to interpret more easily the assumptions, call (*F*_1_,*F*_2_,*M*_1_,*M*_2_) the generic four-dimensional indicator vector, where *F*_1_ and *F*_2_ denote the indicator values at times *t*_1_ and *t*_2_ for females and *M*_1_ and *M*_2_ denote the same quantities for males. The method proceeds according to the following steps.

*Step 1*: central and high scenarios for the first indicator, i.e. *F*_1_, are elicited; of course, these quantities are taken as the mean *μ*(*F*_1_) and the upper quantile *q*(*F*_1_) of *F*_1_ and hence yield its marginal distribution.
*Step 2*: three conditional evaluations are elicited from the expert. To be precise, the expert is required to give

- the central scenario of the indicator for males at time *t*_1_ given that the indicator for females, at the same time, is *μ*(*F*_1_),
- the central scenario of the indicator for males at time *t*_1_ given that the indicator for females, at the same time, is *q*(*F*_1_) and
- the high scenario of the indicator for males at time *t*_1_ given that the indicator for females, at the same time, is *μ*(*F*_1_).

These quantities are taken, in an obvious way, as conditional means or quantiles; in this way, the conditional distribution of *M*_1_ given *F*_1_ is completely defined and then, together with step 1, the joint distribution of (*F*_1_,*M*_1_) is given.

*Step 3*: here the conditional distribution of *F*_2_ given *F*_1_ and *M*_1_ must be defined; we assume, reasonably, that, given *F*_1_, *M*_1_ and *F*_2_ are independent, so we need to fix the distribution of *F*_2_ given *F*_1_. This derives from the following expert-given quantities:

- the central scenario of the indicator for females at time *t*_2_ given that the indicator for females, at time *t*_1_, is *μ*(*F*_1_);
- the central scenario of the indicator for females at time *t*_2_ given that the indicator for females, at time *t*_1_, is *q*(*F*_1_);
- the high scenario of the indicator for females at time *t*_2_ given that the indicator for females, at time *t*_1_, is *μ*(*F*_1_).

These conditions, together with the assumption of independence of *F*_2_ on *M*_1_ given *F*_1_ and the positions in the first two steps, fully characterize the distribution of (*F*_1_,*M*_1_,*F*_2_).

*Step 4*: finally, the conditional distribution of *M*_2_ given *F*_1_, *F*_2_ and *M*_1_ is assumed to depend only on *F*_2_ and *M*_1_ and is then determined by the following conditions:

- the central scenario of the indicator for males at time *t*_2_ given that the indicator for males at time *t*_1_ is *μ*(*M*_1_) and the indicator for females, at time *t*_2_, is *μ*(*F*_2_);
- the central scenario of the indicator for males at time *t*_2_ given that the indicator for males at time *t*_1_ is *μ*(*M*_1_) and the indicator for females, at time *t*_2_, is *q*(*F*_2_);
- the central scenario of the indicator for males at time *t*_2_ given that the indicator for males at time *t*_1_ is *q*(*M*_1_) and the indicator for females, at time *t*_2_, is *μ*(*F*_2_);
- the high scenario of the indicator for males at time *t*_2_ given that the indicator for males at time *t*_1_ is *μ*(*M*_1_) and the indicator for females, at time *t*_2_, is *μ*(*F*_2_).

These four conditions and the assumed independence yield the conditional distribution of *M*_2_ given *F*_1_, *F*_2_ and *M*_1_ and hence, finally, the full joint distribution of the vector (*F*_1_,*F*_2_,*M*_1_,*M*_2_).

Note that, on the whole, we elicit from the expert 12 (conditional) indicator values; these values, together with two (conditional) independence hypotheses, uniquely determine consistently the 14 parameters of the vector under analysis.
